# Magnetotransport study on as-grown and annealed n- and p-type modulation-doped GaInNAs/GaAs strained quantum well structures

**DOI:** 10.1186/1556-276X-9-141

**Published:** 2014-03-24

**Authors:** Ömer Dönmez, Fahrettin Sarcan, Ayse Erol, Mustafa Gunes, Mehmet Çetin Arikan, Janne Puustinen, Mircea Guina

**Affiliations:** 1 Department of Physics, Faculty of Science, Istanbul University, Vezneciler, Istanbul 34134, Turkey; 2 Material Engineering, Adana Science and Technology University, Seyhan, Adana 01180, Turkey; 3 Optoelectronics Research Centre, Tampere University of Technology, Korkeakoulunkatu, Tampere 33720, Finland

**Keywords:** GaInNAs, Magnetotransport, Shubnikov de Haas, Transport, Nitrogen-dependent effective mass

## Abstract

We report the observation of thermal annealing- and nitrogen-induced effects on electronic transport properties of as-grown and annealed n- and p-type modulation-doped Ga_1 - *x*_In_*x*_N_*y*_As_1 - *y*_ (*x =* 0.32, *y* = 0, 0.009, and 0.012) strained quantum well (QW) structures using magnetotransport measurements. Strong and well-resolved Shubnikov de Haas (SdH) oscillations are observed at magnetic fields as low as 3 T and persist to temperatures as high as 20 K, which are used to determine effective mass, 2D carrier density, and Fermi energy. The analysis of temperature dependence of SdH oscillations revealed that the electron mass enhances with increasing nitrogen content. Furthermore, even the current theory of dilute nitrides does not predict a change in hole effective mass; nitrogen dependency of hole effective mass is found and attributed to both strain- and confinement-induced effects on the valence band. Both electron and hole effective masses are changed after thermal annealing process. Although all samples were doped with the same density, the presence of nitrogen in n-type material gives rise to an enhancement in the 2D electron density compared to the 2D hole density as a result of enhanced effective mass due to the effect of nitrogen on conduction band. Our results reveal that effective mass and 2D carrier density can be tailored by nitrogen composition and thermal annealing-induced effects.

**PACS:**

72.00.00; 72.15.Gd; 72.80.Ey

## Review

### Background

Dilute nitrides are technologically important materials due to their promising physical properties and potential application in optoelectronic technology. The strong nitrogen dependence of the bandgap energy makes dilute nitrides promising candidate for device applications, operating in near infrared region [1-3]. Therefore, in order to fully determine fundamental physical properties of this unconventional alloy system, an intense research has been devoted since its discovery. Much effort has been spent developing theoretical models and understanding peculiar nitrogen-induced effects on optical properties of dilute nitrides [1,4-6]. Although the strong composition dependence of the bandgap energy compared to the conventional III-V alloys is attractive, it has been soon realized that the presence of nitrogen severely degrades the optical quality. Therefore, thermal annealing is commonly used a standard procedure to improve the optical quality of dilute nitrides, but at the expense of the blueshift of the bandgap [1,7].

From the electronic properties' point of view, it has been demonstrated that incorporation of nitrogen gives rise to drastic decrease in electron mobility due to the N-induced scattering centers and enhanced electron effective mass [8-13]. On the contrary, in the presence of the nitrogen, it has been theoretically demonstrated that hole effective mass and hole mobility remain unaffected [14-16]. So far, much effort has been focused on nitrogen dependence of electron effective mass and electron mobility, ignoring the composition dependence of hole effective mass and hole mobility. Moreover, even it has been accepted as a standard procedure to improve optical quality, the effects of thermal annealing on electronic properties has not been considered.

The aim of the study presented here is to investigate the effect of nitrogen composition and thermal annealing on electronic transport properties of n- and p-type modulation-doped Ga_0.68_In_0.32_N_*y*_As_1 *- y*_/GaAs (*y =* 0, 0.009, and 0.012) strained quantum well (QW) structures.

### Methods

The samples were grown on semi-insulating GaAs (100) substrates using solid source molecular beam epitaxy, equipped with a radio frequency plasma source for nitrogen incorporation. XRD measurements were used to determine nitrogen and indium compositions. The sample structures are comprised of 7.5-nm-thick QW with indium concentration of 32% and various nitrogen concentration (N% = 0, 0.9, and 1.2) and 20 nm doped (Be for p-type and Si for n-type) GaAs barriers. A 5-nm GaAs was used between GaInNAs and GaAs layer to separate charge and doping regions. The growth temperatures of GaInNAs, GaInAs, and GaAs were 420°C, 540°C, and 580°C, respectively. Post growth rapid thermal annealing was applied at 700°C for 60 and 600 s. The doping density was the same for both n- and p-type samples as 1 × 10^18^ cm^-3^. The samples were fabricated in Hall bar shapes, and ohmic contacts were formed by alloying Au/Ge/Ni and Au/Zn for n- and p-type samples, respectively.

Magnetotransport measurements were carried out using a ^4^He cryostat equipped with a 7 T superconducting magnet. In-plane effective mass, 2D carrier density, and Fermi energy were determined by analyzing the Shubnikov de Haas (SdH) oscillations as a function of temperature between 6.1 and 20 K. In order to evaluate the obtained results from SdH analysis, influence of nitrogen and thermal annealing on the bandgap was probed using photoluminescence (PL) measurements. PL was excited with an argon ion laser (514 nm), dispersed with a 0.5-m monochromator and detected with a thermo-cooled GaInAs photodetector.

### Results and discussion

**Figure 1:**
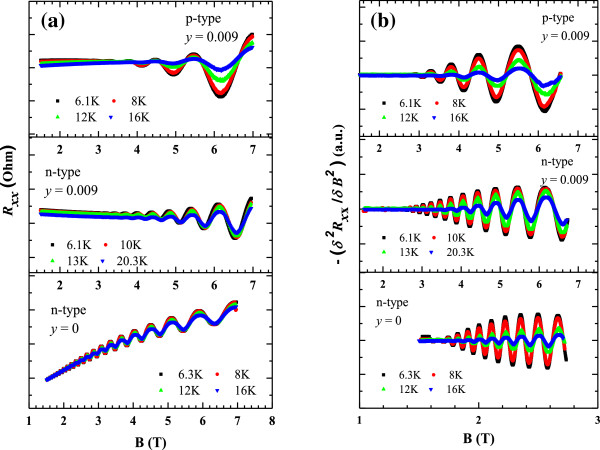
**SdH oscillations. (a)** Raw experimental magnetoresistance data and **(b)** second derivative of the SdH oscillations at different temperatures for the as-grown N-free (*y* = 0) and N-containing (*y =* 0.009) samples.

Figure 1a shows the experimental data of magnetoresistance measurements at various temperatures for one set of the N-containing and N-free as-grown samples. It is known that SdH oscillations can be observed in high magnetic fields (*μB* > 1) in low mobility samples and at low temperatures (*k*_B_*T* < ℏ*ω*_*C*_). Since doping amount is the same in all samples, carrier mobility is an important factor to be able to observe SdH oscillations. As seen in Figure 1, the SdH oscillations start at lower magnetic fields for N-free samples as an indication of higher carrier mobility in N-free samples. It is worth noting that we observed higher mobility in N-free samples in a previous work (see [8]).

The observed decrease of the amplitude of SdH oscillations with increasing temperature can be expressed by an analytical function [17-19]:

(1) $$\frac{\Delta \rho_{\mathrm{xx}}}{\rho_{0}}\propto \sum_{i=\;1}^{n}\exp\;\left(-\frac{\pi }{\omega_{c}\tau_{q}}\right)\cos\;\left[\frac{2\pi i\;\left(E_{F}-E_{1}\right)}{\hbar \omega_{c}}-\mathrm{i\pi}\right]\;D\left(i,\chi \right)$$

(2) $$D\left(\chi \right)=\frac{\chi }{\sinh\chi }\;$$

(3) $$\chi =\frac{2\pi^{2}k_{B}T}{\hbar \omega_{c}}$$

(4) $$\mu_{q}=\frac{e\tau_{q}}{m*}\;$$

(5) $$\omega_{C}=\frac{\mathrm{eB}}{m^{*}}$$

where *Δρ*_*xx*_,  *ρ*_0_,  *E*_F_,  *E*_1_,  *ω*_*c*_,  *m* *,  *τ*_*q*_, and *μ*_*q*_ are the oscillatory magnetoresistivity, zero-field resistivity, Fermi energy, first subband energy, cyclotron frequency, effective mass, quantum lifetime of 2D carriers, and carrier mobility, respectively. The *i* represents the subbands. In Equation 1, the temperature dependence of the amplitude of the oscillations is included in the function *D*(*χ*). The exponential function in Equation 1 represents the damping of the oscillations due to the collision-induced broadening of Landau levels. The contribution of the higher subbands appears in SdH oscillations with different periodicity. We observed that the SdH oscillations has only one period, indicating that only the lowest subband is occupied. The observation of diminishing minima is an indication of absence of background magnetoresistance and presence of 2D carrier gas.

As seen in Figure 1a, the SdH oscillations are suppressed by either a positive (for N-free sample) or a negative (especially for n-type N-containing sample) background magnetoresistance. The minima of SdH oscillations decrease as the magnetic field increases for p-type N-containing samples due to negligible negative magnetoresistance than that of n-type sample. As for N-free samples, a pronounced positive magnetoresistance causes minima to increase with the magnetic field. The origin of the positive magnetoresistance is parallel conduction due to undepleted carriers in barrier layer, herein GaAs. On the other hand, the weak localization effect leads to negative magnetoresistance [19,20]. The background magnetotransport makes the analysis of SdH oscillations difficult especially at low magnetic fields and high temperatures. In order to exclude the effect of the background magnetoresistance and to extract the SdH oscillations, we used the negative second derivative with respect to the magnetic field of raw magnetoresistance data (-∂^2^*R*_*xx*_/∂*B*^2^) (see Figure 1b). As can be easily seen from Equation 1, this method does not change the position of the peak or period of the oscillations and enables to subtract the slowly changing background magnetoresistance and amplifies the short-period oscillations [18,19] as depicted in Figure 1b.

**Figure 2:**
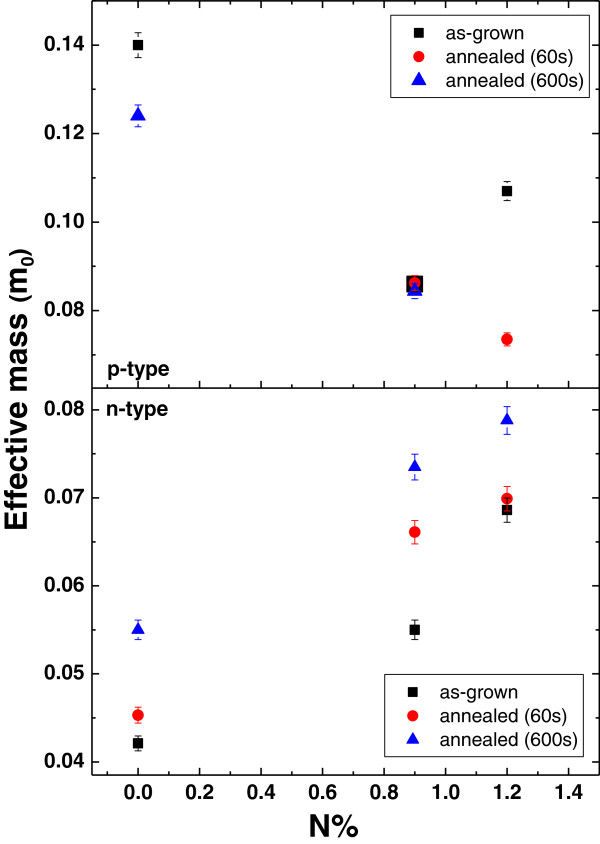
Effective mass values calculated using temperature dependence of SdH oscillations

The thermal damping of the SdH oscillations at a fixed magnetic field is determined by temperature, magnetic field, and effective mass using Equations 1 to 5 as follows [19-22]:

(6) $$\frac{A\left(T,B_{n}\right)}{A\left(T_{0},B_{n}\right)}=\frac{T\sinh\left(2\pi^{2}k_{B}T_{0}m^{*}/\mathrm{\hbar e}B_{n}\right)}{T_{0}\sinh\left(2\pi^{2}k_{B}Tm^{*}/\mathrm{\hbar e}B_{n}\right)}$$

where *A*(*T*, *B*_*n*_) and *A*(*T*_0_, *B*_*n*_) are the amplitudes of the SdH oscillations at a constant magnetic field *B*_*n*_ and at temperatures *T* and *T*_0_. Using Equation 6 and SdH oscillations data at different temperatures, we derived the effective mass which we plotted in Figure 2.

An enhancement of the electron effective mass compared to the N-free sample is observed in N-containing as-grown samples, which obeys the band anti-crossing (BAC) model [4]. After thermal annealing, the electron effective mass increases, which can be attributed to the change of bandgap. It is known that incorporation of nitrogen into GaInAs lattice causes a redshift of the bandgap; on the other hand, thermal annealing blueshifts the bandgap and the amount of blueshift increases with increasing nitrogen content (see Table 1). The origin of the blueshift has been explained in terms of inter-diffusion of In-Ga and restructure of the nearest neighbor configuration of nitrogen [1,9].

**Table 1:** PL peak energies and observed blueshift amounts at 30 K

**Samples**		**PL peak energy (eV)**		**Blueshift (meV)**	
		**p-type**	**n-type**	**p-type**	**n-type**
Ga_0.68_In_0.32_As	As-grown	1.180	1.172	-	-
	Annealed (60 s)	1.182	1.184	2	12
	Annealed (600 s)	1.194	1.194	14	22
Ga_0.682_In_0.32_ N_0.009_As_0.991_	As-grown	1.089	1.120	-	-
	Annealed (60 s)	1.118	1.129	29	9
	Annealed (600 s)	1.146	1.137	57	17
Ga_0.68_In_0.32_ N_0.012_As_0.988_	As-grown	1.033	1.076	-	-
	Annealed (60 s)	1.065	1.088	32	12
	Annealed (600 s)	1.103	1.096	70	20

As a result of blueshift of the bandgap, conduction band states approaches localized N level, giving rise a stronger interaction; therefore, electron effective mass increases compared to the values in as-grown N-containing samples. In N-free sample, indium atoms diffuse out from the QW, leading to a decrease in In content and weaker confinement due to the reduction of the conduction band offset as a result of blueshifted bandgap. An enhancement in electron effective mass in compressively strained GaInAs layer with decreasing In content and weaker confinement was also observed by Meyer et al. [23], which is consistent with our result. As for hole masses, although BAC model does not predict a change in effective hole mass, a decrease with increasing N content from *y* = 0 to *y* = 0.009 resulted in a decrease in hole effective mass. In order to understand the unpredicted N dependence of hole effective mass, both compressive strain- and confinement-induced effects should be considered. With increasing N content, compressive strain decreases and confinement becomes stronger due to the redshift of the bandgap. Stronger confinement decreases the hole effective mass, while less compressive strain increases the hole mass. Moreover, a reduction of the hole concentration decreases the hole effective mass due to change of the valence band non-parabolicity. Therefore, the value of hole effective mass depends on several competing mechanisms. We can conclude that in our N-containing samples, stronger confinement and reduced 2D hole density (see Table 2) are the dominant mechanisms, affecting hole effective mass. A more detailed study of N dependency of hole effective mass and effect of thermal annealing on hole effective mass in these samples can be found in our previous paper [14].

**Table 2:** Effective mass, 2D carrier density, and Fermi energy values found from analysis of SdH oscillations

**Samples**		***n***_**2D **_**(×10**^**12**^ **cm**^**-2**^**)**		**(*E***_**F**_**-E**_**1**_**) (meV)**	
		**p-type**	**n-type**	**p-type**	**n-type**
Ga_0.62_In_0.38_As	As-grown	1.38	2.02	36.8	113.8
	Annealed (60 s)	1.34	1.95	41.5	101.7
	Annealed (600 s)	-	1.92	-	90.9
Ga_0.62_In_0.38_ N_0.009_As_0.991_	As-grown	1.18	2.30	52.7	99.5
	Annealed (60 s)	1.16	2.29	52.0	82.1
	Annealed (600 s)	1.17	2.32	52.8	83.1
Ga_0.62_In_0.38_ N_0.012_As_0.988_	As-grown	1.20	2.50	40.0	0.0686
	Annealed (60 s)	1.06	2.59	55.5	0.0699
	Annealed (600 s)	-	2.71	-	0.0788

**Figure 3:**
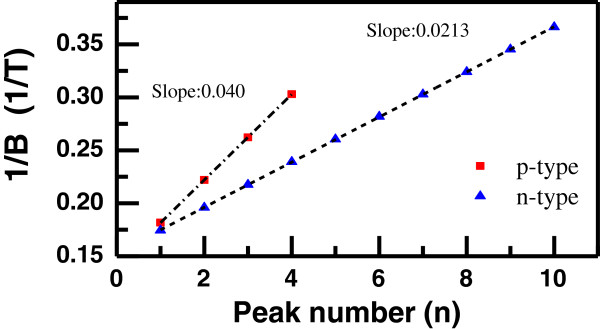
**Plot of 1/*B***_***i***_**versus *n *and the slope of the lines for n- and p-type samples.** The reciprocal magnetic field (1/*B*) versus peak number (*n*) of SdH oscillations for as-grown p- and n-type samples with *y* = 0.009.

The analysis of SdH is also useful to obtain both 2D carrier density and Fermi energy. A plot of the reciprocal magnetic field versus the peak number *n* gives the period of the SdH oscillations, Δ(1/*B*). The 2D carrier density and the Fermi energy can be calculated from the obtained period of SdH oscillations using [18,22,24]

(7) $$\Delta \left(\frac{1}{B}\right)=\frac{\mathrm{e\hbar}}{m^{*}\left(E_{F}-E_{1}\right)}=\frac{e}{\pi \hbar n_{2D}}$$

where *E*_F_ - *E*_1_ is the energy difference between the Fermi level and occupied first subband level; m*, effective mass; and *n*_2D_, 2D carrier density. Figure 3 shows the plot of 1/*B*_*i*_ versus *n* and the slope of the lines for n- and p-type samples with 0.9% nitrogen composition. The fact that the plots have the same slope is an indication of only one occupied subband. We obtained that slopes are independent of temperature. Using the slope of the plot, both 2D carrier density and Fermi energy are calculated and tabulated in Table 2.

Although all samples were doped with the same doping concentration, among n-type samples, among n-type samples, N-free ones have the lowest electron density. Moreover, the hole density is less than the electron density for the samples with the same nitrogen content. An enhancement of electron concentration in N-containing samples compared to the N-free ones was also observed in previous studies [8,14-16] and explained in accordance with the BAC model, since N-induced flattening of conduction band leads to an increased density of states of electrons therefore a significant increase in 2D electron density. Upon thermal annealing, 2D electron density tends to increase in N-containing samples as a result of enhanced electron effective mass. As a result of almost thermal annealing insensitive effective hole mass, 2D hole density remains unaffected for the sample with 0.9% nitrogen. As nitrogen composition increases to 1.2%, the observed decrease in effective hole mass causes to reduce 2D hole density. The calculated Fermi energies change depending on both 2D carrier and effective mass, which are influenced by nitrogen composition and thermal-annealing-induced effects.

## Conclusions

We have investigated the effect of nitrogen and thermal annealing on electronic transport properties of n- and p-type N-free and N-containing alloys using magnetotransport measurements. With an analysis of SdH oscillations at different temperatures, we have calculated in-plane effective carrier mass, 2D carrier density, and Fermi energy of the samples. Nitrogen-dependent enhancement of the both electron and hole masses has been observed in as-grown samples. Upon thermal annealing, the electron effective mass increased, whereas hole mass tends to decrease. The observed nitrogen dependence of electron mass has been explained in terms of strengthened interaction between localized nitrogen level and conduction band states. A tendency to decrease in hole mass upon annealing can be attributed to the reduction of well width and/or decrease in hole density. Even all samples have the same dopant density, the observation of higher 2D electron density than that of p-type samples with the same nitrogen composition and N-free samples has been explained with a stronger interaction of N level and conduction band states, which gives rise to enhancement of the density of states. The results revealed that effective mass in dilute nitride alloys can be tailored by nitrogen composition and also thermal-annealing-induced effects.

## Abbreviations

2D: two-dimensional; QW: quantum well; SdH: Shubnikov de Haas.

## Competing interests

The authors declare that they have no competing interests.

## Authors' contributions

ÖD and FS carried out the experiments and contributed to the writing of the article. AE designed the structure of the samples, conducted the experimental work, and wrote the most part of the article. MG (Adana Science and Technology University) fabricated the samples and contributed to the magnetotransport measurements. MCA supervised the experimental work. JP and MG (Tampere University of Technology) grew and annealed the samples. All authors read and approved the final manuscript.
